# Short- and long-term clinical outcomes of use of beta-interferon or glatiramer acetate for people with clinically isolated syndrome: a systematic review of randomised controlled trials and network meta-analysis

**DOI:** 10.1007/s00415-018-8752-8

**Published:** 2018-01-22

**Authors:** X. Armoiry, A. Kan, G. J. Melendez-Torres, R. Court, P. Sutcliffe, P. Auguste, J. Madan, C. Counsell, A. Clarke

**Affiliations:** 1 Warwick Medical School, Division of Health Sciences, University of Warwick, Gibbet Hill Road, CV4 7AL Coventry, England UK; 2 Division of Applied Health Sciences, University of Aberdeen, Aberdeen, Scotland UK

**Keywords:** Multiple sclerosis, Clinically isolated syndrome, Beta-interferon, Glatiramer acetate, Systematic review

## Abstract

**Background:**

Beta-interferon (IFN-β) and glatiramer acetate (GA) have been evaluated in people with clinically isolated syndrome (CIS) with the aim to delay a second clinical attack and a diagnosis of clinically definite multiple sclerosis (CDMS). We systematically reviewed trials evaluating the short- and long-term clinical effectiveness of these drugs in CIS.

**Methods:**

We searched multiple electronic databases. We selected randomised controlled studies (RCTs) conducted in CIS patients and where the interventions were IFN-β and GA. Main outcomes were time to CDMS, and discontinuation due to adverse events (AE). We compared interventions using random-effect network meta-analyses (NMA). We also reported outcomes from long-term open-label extension (OLE) studies.

**Results:**

We identified five primary studies. Four had open-label extensions following double-blind periods comparing outcomes between early vs delayed DMT. Short-term clinical results (double-blind period) showed that all drugs delayed CDMS compared to placebo. Indirect comparisons did not suggest superiority of any one active drug over another. We could not undertake a NMA for discontinuation due to AE. Long-term clinical results (OLE studies) showed that the risk of developing CDMS was consistently reduced across studies after early DMT treatment compared to delayed DMT (HR = 0.64, 95% CI 0.55, 0.74). No data supported the benefit of DMTs in reducing the time to, and magnitude of, disability progression.

**Conclusions:**

Meta-analyses confirmed that IFN-β and GA delay time to CDMS compared to placebo. In the absence of evidence that early DMTs can reduce disability progression, future research is needed to better identify patients most likely to benefit from long-term DMTs.

**Electronic supplementary material:**

The online version of this article (10.1007/s00415-018-8752-8) contains supplementary material, which is available to authorized users.

## Introduction

Relapsing–remitting multiple sclerosis (RRMS) often originates from a single demyelinating event, known as clinically isolated syndrome (CIS) [1]. CIS events are isolated events of symptomatic neurological disturbance lasting more than 24 h, which indicate the first clinical demyelination of the central nervous system [2]. Clinical syndromes are usually mono-focal but occasionally multi-focal in nature. Based on the previous diagnostic classifications of multiple sclerosis (MS) [3, 4], people with CIS could be categorised as high risk of developing MS but without being formally diagnosed with MS. With the revisions in the magnetic resonance imaging (MRI) component of the 2010 McDonald criteria [5], it is now possible to be diagnosed with MS after CIS if repeated MRI imaging shows multiple demyelinating lesions of the central nervous system developing over time, which has lead to an increased rate of MS diagnosis in patients with CIS [6].

Disease-modifying therapies (DMTs) are commonly prescribed for people diagnosed as having MS. Most randomised controlled trials (RCTs) conducted in RRMS have sought to include patients with at least two acute clinical exacerbations in the prior years. First-generation DMTs, such as beta-interferon (IFN-β) and glatiramer acetate (GA), have also been evaluated in people with CIS with the aim of delaying a second clinical attack leading to the diagnosis of clinically definite MS (CDMS).

In 2008, a systematic review assessed these two types of DMTs in this specific population [7] but newer evidence has become available since then. Moreover, this systematic review assessed the effectiveness of DMTs over a short follow-up duration (2–3 years), which is insufficient to evaluate the long-term clinical effectiveness of DMTs over placebo. This is of particular importance when accounting for the frequent occurrence of adverse events with first-generation DMTs. For example, flu-like symptoms occur in 40–75% of patients with IFN-β, and injection-site reactions and pain are reported by at least one-third of patients with IFN-β or GA [8]. These adverse events can affect the quality of life of patients who may otherwise have only limited disability at this very early disease stage. While an RCT comparing an early to a pre-planned delayed initiation of DMTs would be the best study design to investigate the optimal treatment sequence after CIS, the long-term benefit of early DMTs can be partially assessed from the open-label extension phases of studies which originally compared DMTs to placebo. Thus, in this systematic review and network meta-analysis (NMA), we examined the short- and long-term clinical effectiveness of first-generation DMTs in people with CIS.

## Methods

### Search method

The current work follows a systematic review (PROSPERO identifier: CRD42016043278) that was undertaken as part of a larger project assessing the clinical and cost effectiveness of IFN-β and GA acetate for MS within the UK [8]. The following electronic databases were searched for RCTs in CIS in February 2016: Cochrane Multiple Sclerosis and Rare Diseases of the CNS group specialised register; MEDLINE (Ovid); MEDLINE In-Process and Other Non-Indexed Citations (Ovid); Embase (Ovid); Cochrane Library (Wiley), including Cochrane Database of Systematic Reviews, Cochrane Central Register of Controlled Trials (CENTRAL), DARE, NHS EED, and HTA databases); Science Citation Index and Conference Proceedings—Science (Web of Science); UKCRN Portfolio Database; WHO ICTRP; Current Controlled Trials; ClinicalTrials.gov. No date restriction was applied. The included and excluded study lists from previous relevant Cochrane systematic reviews were also checked [9–11]. Additionally, any CIS RCTs identified during the broader MS systematic review were also considered for inclusion. These additional searches are described in the report of this larger project [8] (Online resource 1). During our original work, we also identified long-term follow-up studies of immediate versus delayed treatment at title/abstract level, but excluded them at full-text review. For the purpose of the current review, we retrieved these records from our initial list of excluded references and undertook targeted searches using more than one source to find other potential subsequent reports from the original CIS studies.

### Selection criteria

Our original systematic review included RCTs conducted in people with RRMS, SPMS, or CIS, evaluating all forms of beta-interferon (IFN β-1a; pegylated IFN β-1a; IFN β-1b) and GA compared against each other or placebo/best supportive care, and where clinical outcomes were reported such as relapse rates, progression to multiple sclerosis, or disability progression as measured by the Expanded Disability Status Scale (EDSS). We consider here a subset of studies reporting RCTs conducted in people with a single clinical attack diagnosed as having CIS and where the interventions were used at the authorised dose regimen (IFN β-1b 250 µg subcutaneously (SC) every other day for Betaferon^®^ (Bayer, Leverkusen, Germany) and Extavia^®^ (Novartis, Basel, Switzerland); IFN β-1a 30 µg intramuscularly (IM) once weekly for Avonex^®^ (Biogen Idec Ltd, Cambridge, MA, USA); GA 20 mg SC daily for Copaxone^®^ (Teva Pharmaceutical Industries, Petah Tikva, Israel); IFN β-1a 44 µg SC three times weekly for Rebif^®^ (Merck, Darmstadt, Germany)).

Our main outcomes of interest were: (1) time to CDMS using Poser criteria [12] and involving a second relapse or neurological deterioration, measured using hazard ratios (HR); we also undertook a sensitivity analysis examining time to ‘McDonald MS’ instead of time to CDMS, in which MRI findings could be used with clinical findings to arrive at a diagnosis; (2) discontinuation due to AE, measured using risk ratios (RR). Although these were not included in our original systematic review, we also included long-term open-label extension studies following the double-blind period of RCTs that had enrolled patients with CIS. Open-label extension studies were defined as studies that compared all those randomised to active treatment who continued on treatment in extension phase (early DMT) versus all those randomised initially to placebo who then crossed over to active treatment in extension phase (delayed DMT). We used these studies to explore the impact of DMTs on disability progression, which was measured using the Expanded Disability Status Scale (EDSS). We also examined long-term annualised relapse rates (ARR) from open-label studies.

### Study selection process

In our initial work, we first examined relevant past systematic reviews from the Cochrane MS group [7, 10, 11] for studies meeting the inclusion criteria. For updated and new searches, we collected all retrieved records in a specialised database and duplicate records were identified and removed. Subsequently, two reviewers applied the inclusion/exclusion criteria and screened all identified bibliographic records for title/abstract and then for full text. Any disagreements were resolved through discussion consensus or by recourse to a third-party reviewer.

### Data extraction, quality assessment, and synthesis

Data extraction was performed by two reviewers and was cross-checked. Any disagreement was resolved through discussion or recourse to a third reviewer. We undertook the quality assessment of included studies using the Cochrane risk of Bias tool for randomised-controlled trials [13]. The quality assessment was cross-checked within the reviewer team. Any disagreements were resolved as in earlier stages. The included papers were organised in text and tables. The summary tables tabulated characteristics and results for included primary studies. Tables for primary studies present summary data on participants, interventions, and outcomes.

For RCTs reported up to the end of double-blind periods, we performed a conventional pairwise meta-analysis using a method-of-moment random effects model (inverse-variance weighted) to directly compare each pair of interventions for which direct evidence exists, and to compare included DMTs as a class against placebo. We examined these pairwise meta-analyses for heterogeneity, measured as Cochran’s Q and I^2^. We then estimated random effects network meta-analyses in the frequentist framework, using package network in Stata v.14. For each outcome of interest, a network diagram of pairwise direct comparisons between multiple treatments was created. Tests for inconsistency were inapplicable because the network was star shaped. The summary estimates (with 95% CIs) for all pair-wise comparisons were provided in a league table [14]. We aimed to determine the rankings of all treatments (e.g. based on the relative probability of an intervention being superior) with respect to effectiveness and safety outcomes in reference to placebo/standard care and we used the surface under the cumulative ranking curve (SUCRA) [14].

For open-label extension studies, we calculated a pooled estimate of the effect of early DMT, including all DMTs as a class, compared to delayed DMT on time to CDMS (effect again measured as HR) through a method-of-moments random effects meta-analysis. When HR were reported at different follow-up durations, we used the most mature data, i.e. those with the longest follow-up. In these studies, the outcomes were only reported for patients originally randomised to active treatment or placebo, who entered extension phase. Our exploratory work measuring the impact of early DMT on disability progression and examining long-term ARR was summarised narratively.

## Results

### Study identification and characteristics

**Fig. 1:**
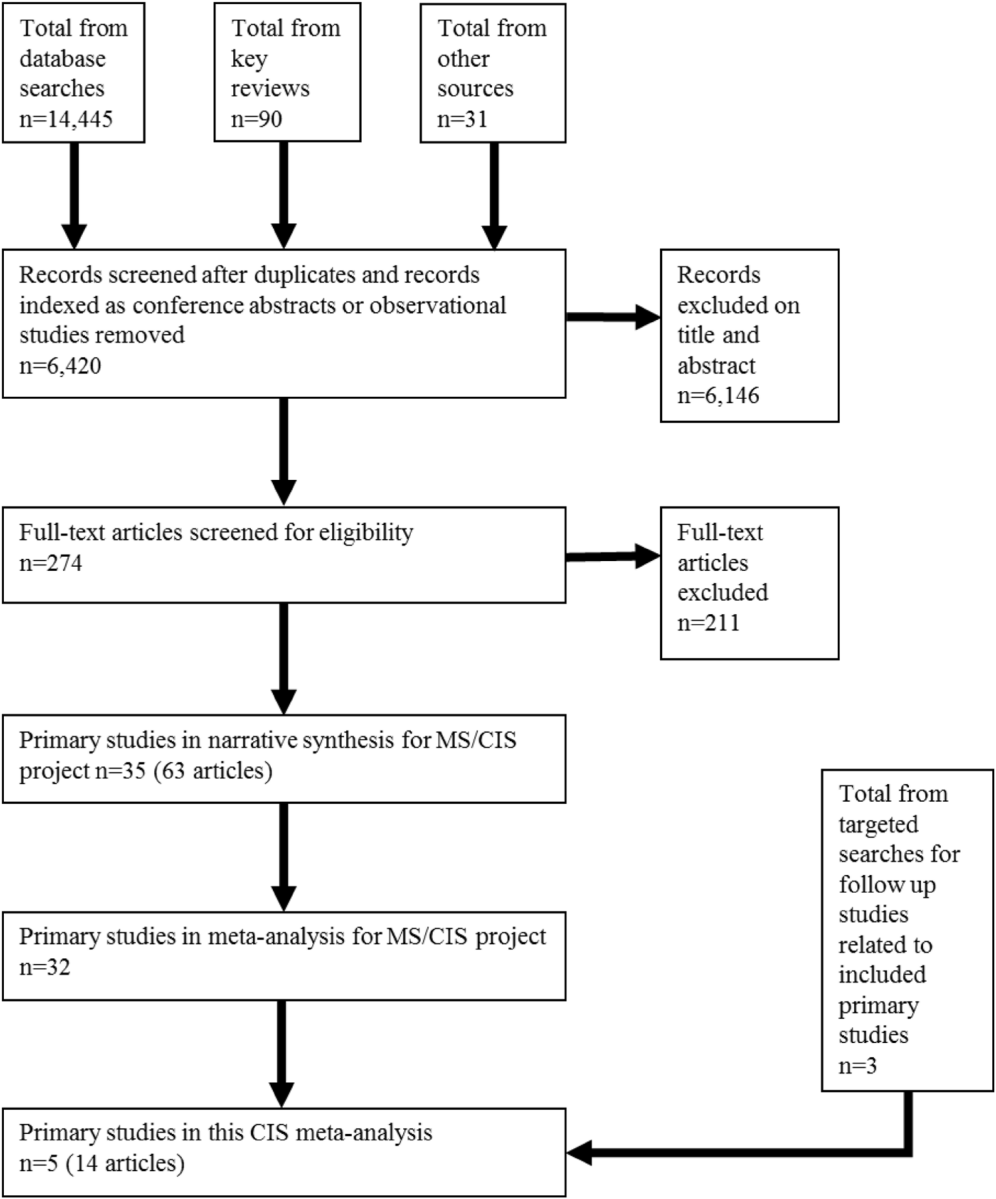
PRISMA flowchart

Our original searches identified 6420 records. Of these, 6146 were excluded based on title and/or abstract, leaving 274 to be examined at full-text (Fig. 1).

Among these, 14 records [15–28] (including two additional ones from our later targeted search) met our inclusion criteria and corresponded to five trials: BENEFIT comparing IFN β-1b 250 µg SC every other day to placebo [15]; CHAMPS [16] and Pakdaman et al. [17] both evaluating IFN β-1a 30 µg IM once a week against placebo; PRECISE comparing the effect of GA 20 mg SC once daily to placebo [18]; and REFLEX comparing IFN β-1a 44 µg SC three times a week against placebo [19]. In these trials, all included patients had a single clinical event and also had evidence of clinically silent lesions based on MRI. The double-blind period in these trials was 2 years after randomisation in BENEFIT and REFLEX, and 3 years in PRECISE, Pakdaman et al. [17], and CHAMPS (Table 1).

**Table 1:** Baseline characteristics of patients in included studies

Study/author, year	Study details	Characteristics of participants at baseline	Intervention (sample size)
BENEFIT 2006	Country: Israel, Canada, and 18 European countries including Germany, Spain, United Kingdom, France, Netherlands, Switzerland Centres: 98 Study period: February 2002 and June 2003. 24 month follow-up Sponsor: Schering AG	Median age: 30 Mean sex: 70.7% female Race: 98.3% white EDSS Score (median): 1.5 Relapse rate: NA Other clinical features of MS: monofocal/plurifocal onset: 52.6/47.4%	Arm 1: IFN β-1b 250 µg SC every other day (*n* = 305) Arm 2: Injections of placebo (*n* = 182)
CHAMPS 2000	Country: USA and Canada Centres: 50 Study period: April 1996 until March 2000. Follow-up 36 months Sponsor: Biogen	Mean age: 33.0 (0.7) Mean sex: 75% female Race: 86% white EDSS Score: NA Relapse rate: NA Other clinical features of MS: Type of initial event: optic neuritis (50%), Spinal cord syndrome (22%), Brainstem or cerebellar syndrome (28%) Type of onset (based on new classification): monofocal, 70%; multifocal, 30% Duration of symptoms before initiation of intravenous methylprednisolone: 8 days Duration of symptoms at initiation of study treatment: 19 days	Arm 1: IFN β-1a 30 µg IM once weekly (*n* = 193) Arm 2: Placebo (*n* = 190)
Pakdaman 2007	Country: Iran Centres: 4 Study period: February 2002 to August 2005. 36 months follow-up Sponsor: unclear	Mean age: 28.0 Mean sex: 67.8% female Race: NA EDSS Score: NA Relapse rate: NA Other clinical features of MS: Type of initial event: optic neuritis 48.0%, spinal cord syndrome 23.8%, brain/cerebellar syndrome 21.8%	Arm 1: IFN β-1a 30 µg IM once weekly (*n* = 104) Arm 2: Injectable placebo (*n* = 98)
PreCISe 2009	Country: Italy, Romania, Argentina, Finland, Austria, Germany, Sweden, Australia, Hungary, France, Norway, Spain, Denmark, Canada, USA, United Kingdom, Centres: 80 Study period: Enrolled from January, 2004, to January, 2006. 36 months follow-up Sponsor: Teva Pharmaceutical Industries	Mean age 31.2 (6.9) Mean sex: 67% FEMALE Race: 96% white EDSS Score: 1.0 (1.0) Relapse rate: NA Other clinical features of MS: Time from first symptom (days): mea*n* = 74.0 (14.1); media*n* = 78.8 (33–104)	Arm 1: GA 20 mg SC daily (*n* = 243) Arm 2: Daily placebo injections (*n* = 238)
REFLEX 2012	Country: Argentina, Austria, Belgium, Bulgaria, Canada, Croatia, Czech Republic, Estonia, Finland, France, Germany, Greece, Israel, Italy, Latvia, Lebanon, Morocco, Poland, Portugal, Romania, Russian Federation, Saudi Arabia, Serbia, Slovakia, Spain, Turkey Centres: 80 Study period: November, 2006 to August, 2010. 24 month double-blind follow-up Sponsor: Merck Serono SA	Mean age: 30.7 Mean sex: 66% female Race: NA EDSS Score: median 1.5 Relapse rate: NA Other clinical features of MS: Time since first demyelinating event (days) = 57.6 Fulfilling McDonald 2010 MS criteria: 37.7% (from Freedman 2014)	Arm 1: IFN β-1a 44 SC three times weekly (*n* = 146) Arm 2: Thrice weekly injections (*n* = 146)

Nine of the 14 selected records were open-label extension studies of four of the five above-listed trials (Online resource 2): BENEFIT with long-term follow-up at 3, 5, 8, and 11 years [20–23]; CHAMPS with results at 5 and 10 years (CHAMPIONS study) [24, 25]; PRECISE with a prolonged follow-up at 5 years [27]; and REFLEX with a 5-year follow-up (REFLEXION study) [26, 28]. We found no subsequent long-term follow-up for the Pakdaman et al. [17] RCT. In open-label extension studies, patients initially allocated to placebo were offered active treatment from the time of a second clinical attack or after the end of the double-blind period (delayed DMT group). Conversely, people initially allocated to active treatment were offered to continue on treatment during an extension study (early DMT group). Overall, the proportion of patients entering open-label extension studies ranged from 53 to 86% after completion of the double-blind phase with no significant differences between the two groups (early or delayed DMTs) within each study (Online resource 2).

We judged there was a substantial proportion of studies with insufficient information to rate the risk of selection bias (60% of studies rated as unclear risk on random sequence generation and 40% of studies rated as unclear risk on allocation concealment) (Online resource 3 and 4). Although studies were placebo controlled, we found that four of the five (80%) studies were at high risk of performance bias (due to loss of blinding of participant and personnel). This was because of the much increased rates of side effects such as injection-site reactions in patients allocated to active treatment which led to high risk of participant unblinding.

Overall, we found no major issue of detection (blinding of outcome assessment) or reporting bias across studies. Only one study [17] was rated as at high risk at attrition bias (incomplete outcome data). Understandably, the open-label extension studies were at much higher risk of attrition bias with a range of complete follow-up rates after entering the extension phase of 44–96%. Finally, all studies funded by drug manufacturers were designated as high risk of bias under the ‘other’ category.

### Short- and long-term clinical outcomes

Short-term clinical outcomes were informed by the results from the double-blind period of RCTs.

#### Time to CDMS: short-term outcomes

**Fig. 2:**
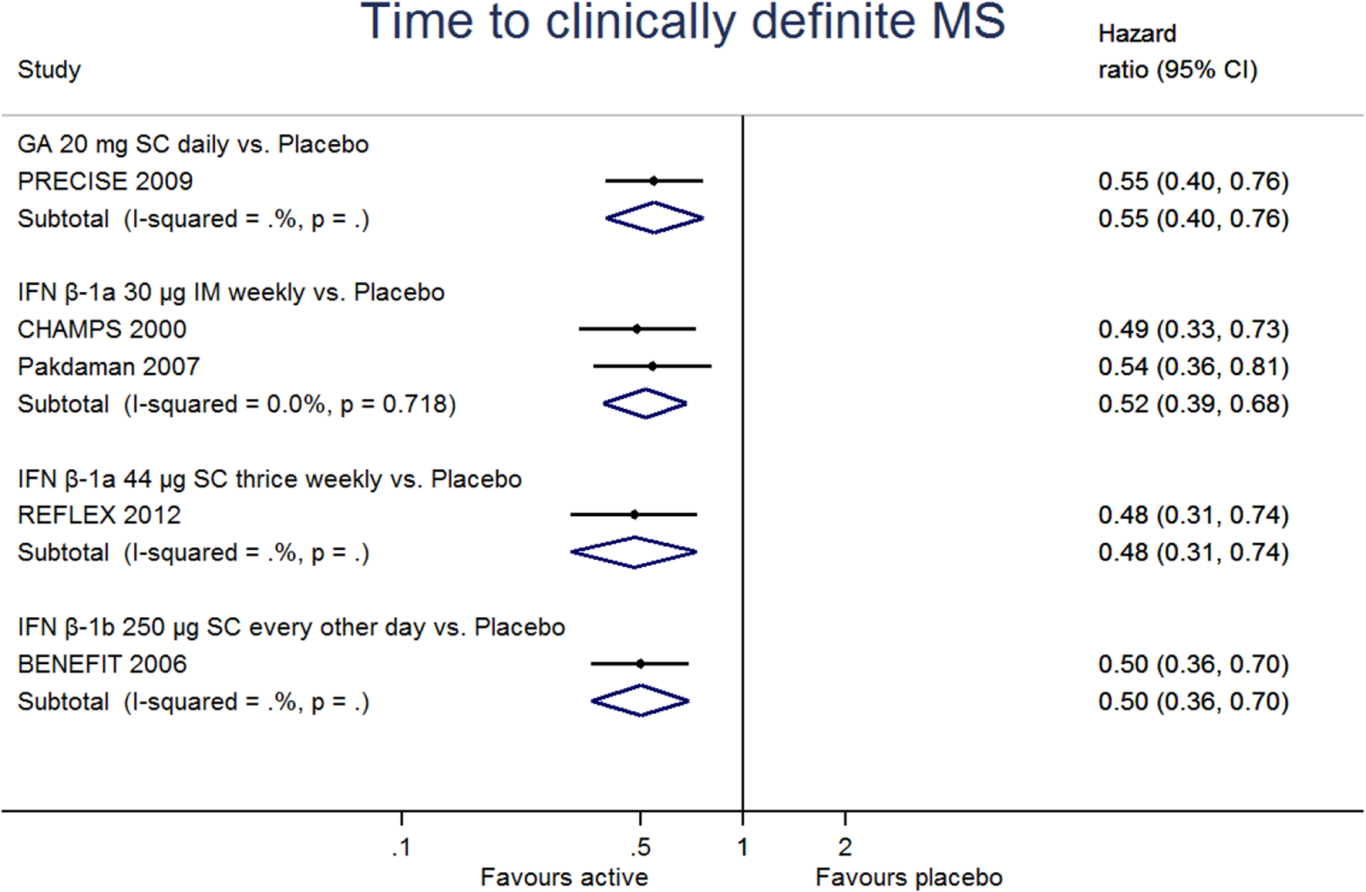
Pairwise meta-analyses, time to clinically definite MS (active drug vs placebo)

Direct evidence from comparisons showed all drugs reduced the time to CDMS compared to placebo (Fig. 2). There was little heterogeneity (*I*^2^ = 0%, *p* = 0.72) for the IFN β-1a 30 μg IM once a week vs. placebo comparison, which was the only one that included more than one study. Accounting for all DMT and considering a class effect for DMTs used in CIS, the pooled HR for time to CDMS was 0.51 (95% CI 0.44, 0.61) with low heterogeneity (*I*^2^ = 0%, *p* = 0.98).

**Fig. 3:**
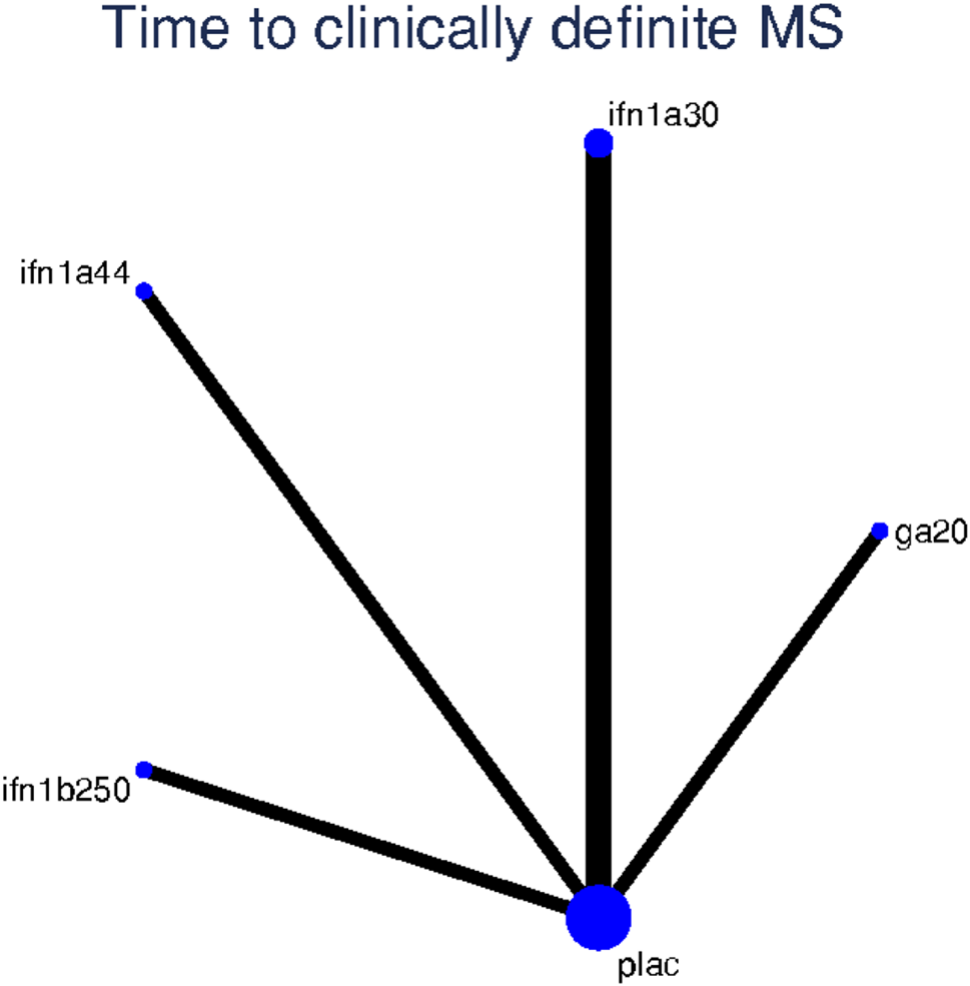
Network of studies, time to clinically definite MS. ifn1a30: IFN β-1a 30 µg IM once a week; ifn1a44: IFN β-1a 44 µg SC three times weekly; ifn1b250: IFN β-1b 250 µg SC every other day; ga20: GA 20 mg SC once daily; plac: placebo

The set of studies reporting hazard ratios for time to CDMS formed a connected, star-shaped, network (Fig. 3). Our random-effect NMA showed similar results compared to pairwise comparisons for active vs placebo comparisons (Table 2). There was no evidence from indirect comparisons suggesting superiority of any one active drug over another. Rankings from the NMA suggested that IFN β-1a 44 μg SC thrice weekly was ranked best, followed by IFN β-1b 250 μg SC every other day, IFN β-1a 30 μg IM once a week and GA 20 mg SC once daily while placebo was ranked last.

**Table 2:** Network meta-analysis: time to CDMS

Drug	SUCRA	IFN β-1a 44 μg SC thrice weekly	IFN β-1b 250 μg SC every other day	IFN β-1a 30 μg IM weekly	Glatiramer 20 mg daily	Placebo
IFN β-1a 44 μg SC thrice weekly	0.70		0.96 (0.56, 1.65)	0.93 (0.56, 1.55)	0.87 (0.51, 1.50)	0.48 (0.31, 0.74)
IFN β-1b 250 μg SC every other day	0.68			0.97 (0.63, 1.50)	0.91 (0.57, 1.45)	0.50 (0.36, 0.70)
IFN β-1a 30 μg IM weekly	0.62				0.94 (0.61, 1.45)	0.52 (0.39, 0.68)
Glatiramer 20 mg daily	0.5					0.55 (0.40, 0.76)
Placebo	0					

Findings are expressed as HR (95% CI)

As a result of our sensitivity analysis, we found that effectiveness estimates were robust to the use of time to McDonald MS instead of time to CDMS [pooled HR for time to ‘McDonald MS’ 0.52 (95% CI 0.46, 0.60); *I*^2^ = 0%; *p* = 0.93].

#### Discontinuation due to AEs: short-term outcomes

All but Pakdaman et al. [17] reported discontinuation due to AEs. However, the time point to assess this outcome was not consistent across the four studies (36 months in PRECISE and CHAMPS; 24 months in REFLEX and BENEFIT). Therefore, we chose not to undertake a NMA for discontinuations due to AE. Estimates from published papers are presented in Table 3 and do not show clear trends across studies.

**Table 3:** Discontinuation due to AEs in CIS studies

Study	Comparison	Follow-up (months)	Treatment arm events	Treatment group	Treatment events proportion (%)	Placebo arm events	Placebo group	Placebo events proportion (%)
PreCISe 2009	GA 20 mg daily vs. placebo	36	14	243	5.8	4	238	1.7
REFLEX 2012	IFN β-1a 44 μg SC thrice weekly vs. placebo	24	5	171	2.9	6	171	3.5
CHAMPS 2000	IFN β-1a 30 μg IM weekly vs. placebo	36	1	193	0.5	7	190	3.7
BENEFIT 2006	IFN β-1b 250 μg SC every other day vs. placebo	24	24	292	8.2	1	176	0.6

#### Time to CDMS: long-term outcomes

Open-label extension studies informed the long-term clinical outcomes. Results presented below include data reported over both original and extension phases (Online resource 2).

At 5 years (3 years of open-label extension in REFLEXION [26] and CHAMPIONS [25]), early IFN β-1a 30 µg IM once weekly led to a delayed time to CDMS compared to the late use of these agents. This was also observed at 10 years (open-label extension in CHAMPIONS [24]) for early IFN β-1a 30 µg IM once weekly. These findings were robust to the use of time to McDonald MS instead of time to CDMS. In CHAMPIONS [24, 25], ARR in patients with delayed DMT was doubled over the 5- and 10-year periods compared to early DMT (0.31 vs 0.14, respectively; *p* = 0.03). At 10 years, 81% of patients had EDSS score ≤ 2.5 and only 6% with EDSS ≥ 6.0, with no significant difference between early and delayed IFN β-1a 30 µg IM once weekly.

In BENEFIT, results from open-label extension follow-up showed a reduced risk of developing CDMS with early IFN β-1b 250 µg SC every other day compared to delayed use of the same DMT for up to 9 years of additional follow-up [20–23]. At 5 years (i.e. including 3 years of open-label extension), risk of EDSS progression in early DMT vs delayed DMT was 25 and 29%, respectively, annualised relapse rates were lower with early IFN β-1b 250 µg SC every other day (0.21 vs 0.27, respectively; *p* = 0.014) while EDSS scores were stable in both groups (− 0.03 compared to baseline with early DMT and + 0.07 compared to baseline with delayed DMT) [23]. At 8 years (6 years of open-label extension), overall ARR was reduced by 22.9% and relapse risk was reduced by 23.4% (HR 0.77, 95% CI 0.589–0.988; *p* = 0.048) with early DMT [20]. EDSS scores at the end of the 8-year period were on average 1.87 with early DMT (+ 0.38 change from baseline) and 1.56 with delayed DMT (+ 0.07 compared to baseline). At 11 years (9 years of open-label extension), overall ARR was lowered with early IFN β-1b vs delayed IFN β-1b (0.21 vs 0.26, respectively, *p* = 0.0018) while EDSS scores had minimal changes remaining low in both groups (mean of 2.04 with early IFN β-1b, + 0.55 compared to baseline; mean of 2.22 with delayed IFN β-1b, + 0.72) [21].

With early GA, the time to CDMS was delayed by 41% (HR 0.59; 95% CI 0.44–0.80) compared to delayed GA at 5 years [27]. ARRs were 0.11 and 0.16, respectively (*p* = 0.0241). EDSS score changes over the 5-year period were minimal.

**Fig. 4:**
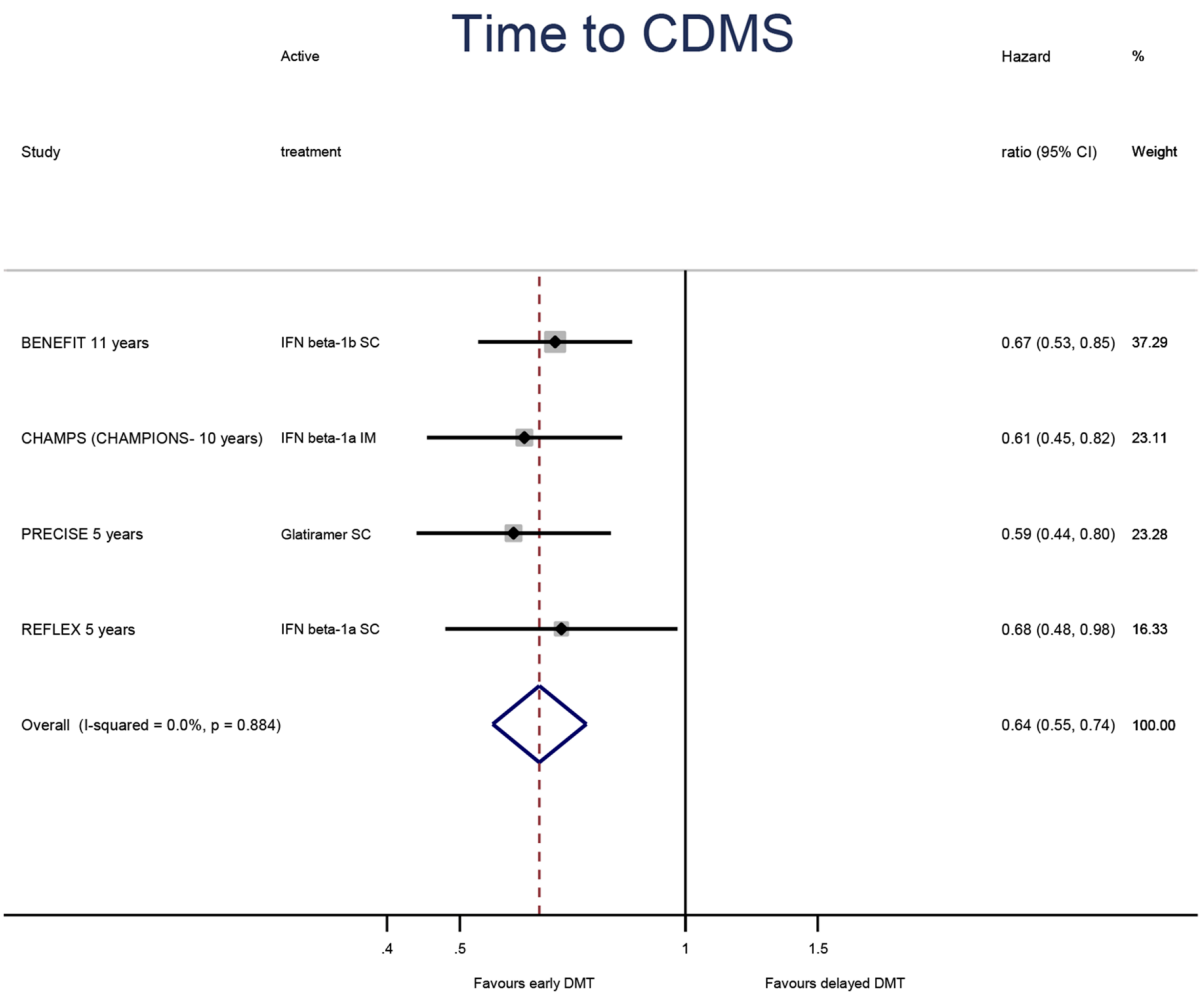
Pooled estimate of time to CDMS (early vs delayed DMT)

Accounting for all DMTs, the pooled HR for time to CDMS was 0.64 (95% CI 0.55, 0.74) with low heterogeneity (*I*^2^ = 0%, *p* = 0.89) (Fig. 4) demonstrating the benefit of early vs delayed DMT.

## Discussion

Meta-analyses confirmed that IFN-β and GA delayed the time to CDMS after CIS compared to placebo. No active drug showed superiority over another. Similar findings were observed in our sensitivity analysis using time to McDonald MS instead of time to CDMS. We were not able to compare drugs in relation to the risk of discontinuation due to AE.

Findings from open-label extension studies suggested a persistent long-term benefit in terms of time to CDMS for earlier treatment, despite the relatively short delay in treatment initiation (2–3 years). However, these results should be treated with caution because of a high-risk of selection bias which may make delayed DMT appear worse than early DMT. The fact that patients initially allocated to placebo were offered active treatment from the time of a second clinical attack, and that the risk of developing CMDS was reduced with DMT compared to placebo over the double-blind period means that those in placebo arms who had a second attack were more likely to get into extension phase. This would result in higher risk of MS in the delayed DMT group than in the early DMT group. Moreover, most of these studies had large losses to follow-up (14–47%), which gives the potential for attrition bias. This makes it difficult to be certain of the long-term benefit in delaying the time to CDMS.

The evidence showing the benefit of IFN-β and GA in delaying time to CDMS after CIS could underline the importance of initiating specific therapies in a timely manner following CIS. However, the decision about whether to start DMTs is more complex. Some patients with CIS will never have a second attack even off treatment. This is suggested by cohort studies reporting high proportions of CIS patients having no second attack despite not receiving DMTs, 55% after a mean follow-up of 5.8 years according to Brownlee et al. [29], or 27% at 10 years in the paper by Kerbrat et al. [30]. Also, some patients with CIS may only have a few minor non-disabling attacks. These patients with benign disease would not gain any/much benefit from treatment but would be exposed to unnecessary risk and/or experience frequent local or systemic adverse events which may reduce their quality of life.

For those who are at high risk of developing more severe MS, there may be some merit in delaying a second attack, but the key aim (from both a clinical and cost-effective perspective) would be to delay irreversible, progressive disability and there is no evidence at present that early treatment after CIS does this. An analogous situation might be whether to use anticonvulsants after a single seizure, given that some patients will never have another seizure. This is generally not recommended, at least in the UK, because the evidence shows early treatment does not improve long-term outcome [31].

The main strength of our work is that we present an up-to-date review on the clinical outcomes of first-generation DMTs, namely IFN-β and GA, used in people with CIS. For that purpose, we used a rigorous and comprehensive search to identify all existing primary studies. This is also the first review to include open-label extension studies, which provide a signal (albeit biased as previously discussed) of the longer term clinical effectiveness of DMTs in this population.

There were several limitations. We were interested in people with CIS and we only included studies where patients had a single clinical attack. It is possible that some of these patients would now meet diagnostic criteria for MS based on new MRI criteria [5], which means that some of these would be considered eligible to begin DMTs. However, given that our main interest was to assess the evidence related to the clinical effectiveness of DMTs in people with a single clinical event we feel this is a minor limitation. To the best of our knowledge, there is no RCT which evaluates the effectiveness of DMTs in people with CIS using the new diagnostic criteria. A second limitation is the inconsistent reporting of safety outcomes, which led to an inability to compare DMTs on discontinuation due to AE. However, we believe that the safety profile of DMTs in CIS can be understood through the results observed in people with RRMS. Indeed, there is no a priori reason why the safety profile of IFN-β and GA would differ in people with CIS compared to those with RRMS because the drugs, strength and dose regimens are similar across both indications. In our work conducted in RRMS based on follow-up at 24 months, we found no evidence that one drug was more likely to lead to discontinuation than other, although confidence intervals for estimates from mixed treatment comparisons were wide. Therefore, we are fairly confident that these findings also apply in CIS patients.

Although we originally reviewed studies reporting long-term open-label extensions of RCTs conducted in people with CIS, this work was conducted post hoc after the original work for our initial systematic review. However, we conducted additional searches for this analysis and are, therefore, reasonably confident that we retrieved all extension studies published as original articles and that no bias was introduced in this subsequent analysis. Finally, a potential limitation of our work, inherent to all systematic reviews, is publication bias since, for instance, some studies finding no impact of DMTs in people with CIS might have remained unpublished. There were too few studies to formally test for this.

In this review, we have focused on first-generation DMTs (IFN-β and GA) since these were all evaluated in CIS-specific RCTs. Oral teriflunomide, a newer generation DMT, has also been tested for patients with CIS in a RCT [32] that reported a benefit over placebo in delaying time to CDMS, yet with the same uncertainty on long-term clinical benefit.

At the European level, we are aware of the 2015 guidelines for prescribing disease-modifying treatments in MS [33]. Our findings agree with these guidelines, stating the benefits of use of the only two DMT types with a licensed indication in CIS in delaying CDMS, namely IFN-β and GA. The Association of British Neurologists (ABN) guidelines also emphasise the uncertainty around the effectiveness of these drugs over a long-term perspective.

In summary, IFN-β and GA reduce the short-term (up to 2–3 years) risk of a second clinical attack after CIS and so delay the diagnosis of CDMS. Open-label extension studies suggest a possible long-term effect of IFN-β and GA in delaying CDMS but there is less certainty because of the risk of bias of these studies. There is currently no evidence supporting the effectiveness of DMTs following CIS in delaying irreversible or progressive disability. Further research with RCTs is needed to specifically assess early vs delayed DMT after CIS to establish the long-term benefit/risk balance of DMTs and whether high-risk patients who might benefit more from early treatment can be identified.

## Electronic supplementary material

Below is the link to the electronic supplementary material.

Supplementary material 1 (DOCX 49 kb)

## References

[1] Confavreux C, Vukusic S (2006). The natural history of multiple sclerosis. Rev Prat.

[2] National Multiple Sclerosis Society Clinically isolated syndrome (CIS) (2015) http://www.nationalmssociety.org/about-multiple-sclerosis/what-we-know-about-ms/diagnosing-ms/cis/index.aspx. Accessed 23 October 2015

[3] McDonald WI, Compston A, Edan G, Goodkin D, Hartung HP, Lublin FD, McFarland HF, Paty DW, Polman CH, Reingold SC, Sandberg-Wollheim M, Sibley W, Thompson A, van den Noort S, Weinshenker BY, Wolinsky JS (2001). Recommended diagnostic criteria for multiple sclerosis: guidelines from the International Panel on the diagnosis of multiple sclerosis. Ann Neurol.

[4] Polman CH, Reingold SC, Edan G, Filippi M, Hartung HP, Kappos L, Lublin FD, Metz LM, McFarland HF, O’Connor PW, Sandberg-Wollheim M, Thompson AJ, Weinshenker BG, Wolinsky JS (2005). Diagnostic criteria for multiple sclerosis: 2005 revisions to the “McDonald Criteria”. Ann Neurol.

[5] Polman CH, Reingold SC, Banwell B, Clanet M, Cohen JA, Filippi M, Fujihara K, Havrdova E, Hutchinson M, Kappos L, Lublin FD, Montalban X, O’Connor P, Sandberg-Wollheim M, Thompson AJ, Waubant E, Weinshenker B, Wolinsky JS (2011). Diagnostic criteria for multiple sclerosis: 2010 revisions to the McDonald criteria. Ann Neurol.

[6] Rosenkranz SC, Kaulen B, Neuhaus A, Siemonsen S, Kopke S, Daumer M, Stellmann JP, Heesen C (2017). Low clinical conversion rate in clinically isolated syndrome (CIS) patients—diagnostic benefit of McDonald 2010 criteria?. Eur J Neurol.

[7] Clerico M, Faggiano F, Palace J, Rice G, Tintore M, Durelli L (2008). Recombinant interferon beta or glatiramer acetate for delaying conversion of the first demyelinating event to multiple sclerosis. Cochrane Database Syst Rev.

[8] Melendez-Torres GJ, Auguste P, Armoiry X, Maheswaran H, Court R, Madan J, Kan A, Lin S, Counsell C, Patterson J, Rodrigues J, Ciccarelli O, Fraser H, Clarke A (2017) Clinical effectiveness and cost-effectiveness of beta-interferon and glatiramer acetate for treating multiple sclerosis: systematic review and economic evaluation. Health Technol Assess (Winch Engl) 21 (52):1–352. 10.3310/hta21520 10.3310/hta21520 PMC5623930 28914229

[9] Clerico M, Faggiano F, Palace J, Rice G, Tintore M, Durelli L (2008). Recombinant interferon beta or glatiramer acetate for delaying conversion of the first demyelinating event to multiple sclerosis. Cochrane Database Syst Rev.

[10] Filippini G, Del Giovane C, Vacchi L, D’Amico R, Di Pietrantonj C, Beecher D, Salanti G (2013). Immunomodulators and immunosuppressants for multiple sclerosis: a network meta-analysis. Cochrane Database Syst Rev.

[11] Tramacere I, Del Giovane C, Salanti G, D’Amico R, Filippini G (2015). Immunomodulators and immunosuppressants for relapsing-remitting multiple sclerosis: a network meta-analysis. Cochrane Database Syst Rev.

[12] Poser CM, Paty DW, Scheinberg L, McDonald WI, Davis FA, Ebers GC, Johnson KP, Sibley WA, Silberberg DH, Tourtellotte WW (1983). New diagnostic criteria for multiple sclerosis: guidelines for research protocols. Ann Neurol.

[13] Higgins JP, Altman DG, Gotzsche PC, Juni P, Moher D, Oxman AD, Savovic J, Schulz KF, Weeks L, Sterne JA (2011). The Cochrane Collaboration’s tool for assessing risk of bias in randomised trials. BMJ.

[14] Jansen JP, Fleurence R, Devine B, Itzler R, Barrett A, Hawkins N, Lee K, Boersma C, Annemans L, Cappelleri JC (2011). Interpreting indirect treatment comparisons and network meta-analysis for health-care decision making: report of the ISPOR Task Force on Indirect Treatment Comparisons Good Research Practices: part 1. Value Health.

[15] Kappos L, Polman CH, Freedman MS, Edan G, Hartung HP, Miller DH, Montalban X, Barkhof F, Bauer L, Jakobs P, Pohl C, Sandbrink R (2006). Treatment with interferon beta-1b delays conversion to clinically definite and McDonald MS in patients with clinically isolated syndromes. Neurology.

[16] Jacobs LD, Beck RW, Simon JH, Kinkel RP, Brownscheidle CM, Murray TJ, Simonian NA, Slasor PJ, Sandrock AW (2000). Intramuscular interferon beta-1a therapy initiated during a first demyelinating event in multiple sclerosis. CHAMPS Study Group. N Engl J Med.

[17] Pakdaman H, Sahraian MA, Fallah A, Pakdaman R, Ghareghozli K, Ghafarpour M, Rahimian E, Shirani A (2007). Effect of early interferon beta-1a therapy on conversion to multiple sclerosis in Iranian patients with a first demyelinating event. Acta Neurol Scand.

[18] Comi G, Martinelli V, Rodegher M, Moiola L, Bajenaru O, Carra A, Elovaara I, Fazekas F, Hartung HP, Hillert J, King J, Komoly S, Lubetzki C, Montalban X, Myhr KM, Ravnborg M, Rieckmann P, Wynn D, Young C, Filippi M, Pre CSG (2009). Effect of glatiramer acetate on conversion to clinically definite multiple sclerosis in patients with clinically isolated syndrome (PreCISe study): a randomised, double-blind, placebo-controlled trial. Lancet.

[19] Comi G, De Stefano N, Freedman MS, Barkhof F, Polman CH, Uitdehaag BMJ, Casset-Semanaz F, Hennessy B, Moraga MS, Rocak S, Stubinski B, Kappos L (2012). Comparison of two dosing frequencies of subcutaneous interferon beta-1a in patients with a first clinical demyelinating event suggestive of multiple sclerosis (REFLEX): a phase 3 randomised controlled trial. Lancet Neurol.

[20] Edan G, Kappos L, Montalban X, Polman CH, Freedman MS, Hartung HP, Miller D, Barkhof F, Herrmann J, Lanius V, Stemper B, Pohl C, Sandbrink R, Pleimes D (2014). Long-term impact of interferon beta-1b in patients with CIS: 8-year follow-up of BENEFIT. J Neurol Neurosurg Psychiatry.

[21] Kappos L, Edan G, Freedman MS, Montalban X, Hartung HP, Hemmer B, Fox EJ, Barkhof F, Schippling S, Schulze A, Pleimes D, Pohl C, Sandbrink R, Suarez G, Wicklein EM (2016). The 11-year long-term follow-up study from the randomized BENEFIT CIS trial. Neurology.

[22] Kappos L, Freedman MS, Polman CH, Edan G, Hartung HP, Miller DH, Montalban X, Barkhof F, Radu EW, Bauer L, Dahms S, Lanius V, Pohl C, Sandbrink R (2007). Effect of early versus delayed interferon beta-1b treatment on disability after a first clinical event suggestive of multiple sclerosis: a 3-year follow-up analysis of the BENEFIT study. Lancet (Lond Engl).

[23] Kappos L, Freedman MS, Polman CH, Edan G, Hartung HP, Miller DH, Montalban X, Barkhof F, Radu EW, Metzig C, Bauer L, Lanius V, Sandbrink R, Pohl C (2009). Long-term effect of early treatment with interferon beta-1b after a first clinical event suggestive of multiple sclerosis: 5-year active treatment extension of the phase 3 BENEFIT trial. Lancet Neurol.

[24] Kinkel RP, Dontchev M, Kollman C, Skaramagas TT, O’Connor PW, Simon JH (2012). Association between immediate initiation of intramuscular interferon beta-1a at the time of a clinically isolated syndrome and long-term outcomes: a 10-year follow-up of the Controlled High-Risk Avonex Multiple Sclerosis Prevention Study in Ongoing Neurological Surveillance. Arch Neurol.

[25] Kinkel RP, Kollman C, O’Connor P, Murray TJ, Simon J, Arnold D, Bakshi R, Weinstock-Gutman B, Brod S, Cooper J, Duquette P, Eggenberger E, Felton W, Fox R, Freedman M, Galetta S, Goodman A, Guarnaccia J, Hashimoto S, Horowitz S, Javerbaum J, Kasper L, Kaufman M, Kerson L, Mass M, Rammohan K, Reiss M, Rolak L, Rose J, Scott T, Selhorst J, Shin R, Smith C, Stuart W, Thurston S, Wall M (2006). IM interferon beta-1a delays definite multiple sclerosis 5 years after a first demyelinating event. Neurology.

[26] Comi G, De Stefano N, Freedman MS, Barkhof F, Uitdehaag BM, de Vos M, Marhardt K, Chen L, Issard D, Kappos L (2017). Subcutaneous interferon beta-1a in the treatment of clinically isolated syndromes: 3-year and 5-year results of the phase III dosing frequency-blind multicentre REFLEXION study. J Neurol Neurosurg Psychiatry.

[27] Comi G, Martinelli V, Rodegher M, Moiola L, Leocani L, Bajenaru O, Carra A, Elovaara I, Fazekas F, Hartung HP, Hillert J, King J, Komoly S, Lubetzki C, Montalban X, Myhr KM, Preziosa P, Ravnborg M, Rieckmann P, Rocca MA, Wynn D, Young C, Filippi M (2013). Effects of early treatment with glatiramer acetate in patients with clinically isolated syndrome. Mult Scler.

[28] Anonymous long-term follow-up of patients who participated in study 27025 (REFLEX) (REFLEXION). https://clinicaltrials.gov/ct2/show/results/NCT00813709?term=reflex+and+ms&rank=2&sect=X10256%20-%20base. Accessed 14 Dec 2017

[29] Brownlee WJ, Swanton JK, Altmann DR, Ciccarelli O, Miller DH (2015). Earlier and more frequent diagnosis of multiple sclerosis using the McDonald criteria. J Neurol Neurosurg Psychiatry.

[30] Kerbrat A, Hamonic S, Leray E, Tron I, Edan G, Yaouanq J (2015). Ten-year prognosis in multiple sclerosis: a better outcome in relapsing-remitting patients but not in primary progressive patients. Eur J Neurol.

[31] Leone MA, Giussani G, Nolan SJ, Marson AG, Beghi E (2016). Immediate antiepileptic drug treatment, versus placebo, deferred, or no treatment for first unprovoked seizure. Cochrane Database Syst Rev.

[32] Miller AE, Wolinsky JS, Kappos L, Comi G, Freedman MS, Olsson TP, Bauer D, Benamor M, Truffinet P, O’Connor PW (2014). Oral teriflunomide for patients with a first clinical episode suggestive of multiple sclerosis (TOPIC): a randomised, double-blind, placebo-controlled, phase 3 trial. Lancet Neurol.

[33] Scolding N, Barnes D, Cader S, Chataway J, Chaudhuri A, Coles A, Giovannoni G, Miller D, Rashid W, Schmierer K, Shehu A, Silber E, Young C, Zajicek J (2015). Association of British Neurologists: revised (2015) guidelines for prescribing disease-modifying treatments in multiple sclerosis. Pract Neurol.

