# Supplementary material for: Short- and long-term clinical outcomes of use of beta-interferon or glatiramer acetate for people with clinically isolated syndrome: a systematic review of randomised controlled trials and network meta-analysis
Source: J Neurol. 2018 Jan 22;265(5):999–1009. doi: 10.1007/s00415-018-8752-8 (PMC5937891; doi:10.1007/s00415-018-8752-8)
Supplement: Supplementary file 1 — Supplementary material 1 (DOCX 49 kb) [file 415_2018_8752_MOESM1_ESM.docx]

## Online resource 1: Search strategy

## Clinically Isolated Syndrome searches

### Review articles checked for included studies and studies excluded with reasons

Cochrane Reviews: Clerico 2008

### Medline (Ovid), searched 09/02/2016

Exact database: Ovid MEDLINE(R) 1946 to January Week 4 2016

| 1 | Demyelinating Diseases/ | 10446 |
| --- | --- | --- |
| 2 | Myelitis, Transverse/ | 1153 |
| 3 | exp Optic Neuritis/ | 6737 |
| 4 | Encephalomyelitis, Acute Disseminated/ | 1689 |
| 5 | Demyelinating Autoimmune Diseases, CNS/ | 316 |
| 6 | demyelinating disease*.tw. | 4725 |
| 7 | transverse myelitis.tw. | 1356 |
| 8 | neuromyelitis optica.tw. | 1735 |
| 9 | optic neuritis.tw. | 3792 |
| 10 | acute disseminated encephalomyelitis.tw. | 1098 |
| 11 | devic.tw. | 107 |
| 12 | ADEM.tw. | 574 |
| 13 | demyelinating disorder.tw. | 335 |
| 14 | clinically isolated syndrome.tw. | 644 |
| 15 | first demyelinating event.tw. | 68 |
| 16 | 1 or 2 or 3 or 4 or 5 or 6 or 7 or 8 or 9 or 10 or 11 or 12 or 13 or 14 or 15 | 24564 |
| 17 | randomized controlled trial.pt. | 404260 |
| 18 | (random* or "controlled trial*" or "clinical trial*" or rct).tw. | 875933 |
| 19 | 17 or 18 | 975513 |
| 20 | (metaanalys* or "meta analys*" or "meta-analys*").tw. | 69583 |
| 21 | "systematic* review*".mp. | 61879 |
| 22 | meta analysis.pt. | 60490 |
| 23 | 20 or 21 or 22 | 123386 |
| 24 | 16 and 19 | 661 |
| 25 | 16 and 23 | 74 |
| 26 | 24 or 25 | 713 |

### Medline In-Process & Other Non-Indexed Citations (Ovid), searched 09/02/2016

Actual database: Ovid MEDLINE(R) In-Process & Other Non-Indexed Citations February 08, 2016

| 1 | demyelinating disease*.tw. | 405 |
| --- | --- | --- |
| 2 | transverse myelitis.tw. | 148 |
| 3 | neuromyelitis optica.tw. | 317 |
| 4 | optic neuritis.tw. | 356 |
| 5 | acute disseminated encephalomyelitis.tw. | 128 |
| 6 | devic.tw. | 6 |
| 7 | ADEM.tw. | 83 |
| 8 | demyelinating disorder.tw. | 55 |
| 9 | clinically isolated syndrome.tw. | 115 |
| 10 | first demyelinating event.tw. | 6 |
| 11 | 1 or 2 or 3 or 4 or 5 or 6 or 7 or 8 or 9 or 10 | 1249 |
| 12 | (random* or "controlled trial*" or "clinical trial*" or rct).tw. | 108853 |
| 13 | (metaanalys* or "meta analys*" or "meta-analys*").tw. | 14202 |
| 14 | "systematic* review*".tw. | 15358 |
| 15 | 13 or 14 | 23763 |
| 16 | 11 and 12 | 63 |
| 17 | 11 and 15 | 17 |
| 18 | 16 or 17 | 73 |

### Embase (Ovid), searched 09/02/2016

Actual database: Embase 1974 to 2016 Week 06

| 1 | demyelinating disease/ | 12216 |
| --- | --- | --- |
| 2 | myelitis/ | 6771 |
| 3 | optic neuritis/ | 6979 |
| 4 | acute disseminated encephalomyelitis/ | 1378 |
| 5 | myelooptic neuropathy/ | 4897 |
| 6 | demyelinating disease*.tw. | 7443 |
| 7 | transverse myelitis.tw. | 2462 |
| 8 | neuromyelitis optica.tw. | 4162 |
| 9 | optic neuritis.tw. | 6551 |
| 10 | acute disseminated encephalomyelitis.tw. | 1762 |
| 11 | devic.tw. | 229 |
| 12 | ADEM.tw. | 1211 |
| 13 | demyelinating disorder.tw. | 624 |
| 14 | clinically isolated syndrome.tw. | 1758 |
| 15 | first demyelinating event.tw. | 159 |
| 16 | 1 or 2 or 3 or 4 or 5 or 6 or 7 or 8 or 9 or 10 or 11 or 12 or 13 or 14 or 15 | 34739 |
| 17 | randomized controlled trial/ | 394252 |
| 18 | (random* or "controlled trial*" or "clinical trial*" or rct).tw. | 1311256 |
| 19 | 17 or 18 | 1393301 |
| 20 | meta analysis/ | 103826 |
| 21 | (metaanalys* or "meta analys*" or "meta-analys*").tw. | 111288 |
| 22 | "systematic review"/ | 101172 |
| 23 | "systematic* review*".tw. | 97114 |
| 24 | 20 or 21 or 22 or 23 | 223913 |
| 25 | 16 and 19 | 1706 |
| 26 | 16 and 24 | 322 |
| 27 | 25 or 26 | 1914 |
| 28 | limit 27 to (conference abstract or conference paper or conference proceeding or "conference review") | 493 |
| 29 | 27 not 28 | 1421 |
| 30 | limit 29 to human | 1340 |
| 31 | limit 29 to animals | 59 |
| 32 | 31 not 30 | 59 |
| 33 | 29 not 32 | 1362 |

### Cochrane Library (Wiley), searched 09/02/2016

| ID | Search | Hits |
| --- | --- | --- |
| #1 | MeSH descriptor: [Multiple Sclerosis] explode all trees | 2125 |
| #2 | multiple sclerosis:ti,ab,kw (Word variations have been searched) | 5081 |
| #3 | #1 or #2 | 5081 |
| #4 | first or early or "clinically isolated":ti,ab,kw (Word variations have been searched) | 166444 |
| #5 | #3 and #4 | 1037 |
| #6 | MeSH descriptor: [Demyelinating Diseases] this term only | 71 |
| #7 | MeSH descriptor: [Myelitis, Transverse] this term only | 6 |
| #8 | MeSH descriptor: [Optic Neuritis] explode all trees | 95 |
| #9 | MeSH descriptor: [Encephalomyelitis, Acute Disseminated] this term only | 3 |
| #10 | MeSH descriptor: [Demyelinating Autoimmune Diseases, CNS] this term only | 2 |
| #11 | demyelinating next disease*:ti,ab,kw (Word variations have been searched) | 186 |
| #12 | transverse myelitis:ti,ab,kw (Word variations have been searched) | 14 |
| #13 | neuromyelitis optica:ti,ab,kw (Word variations have been searched) | 20 |
| #14 | optic neuritis:ti,ab,kw (Word variations have been searched) | 220 |
| #15 | acute disseminated encephalomyelitis:ti,ab,kw (Word variations have been searched) | 13 |
| #16 | devic:ti,ab,kw (Word variations have been searched) | 2 |
| #17 | ADEM:ti,ab,kw (Word variations have been searched) | 4 |
| #18 | demyelinating disorder:ti,ab,kw (Word variations have been searched) | 49 |
| #19 | clinically isolated syndrome:ti,ab,kw (Word variations have been searched) | 114 |
| #20 | first demyelinating event:ti,ab,kw (Word variations have been searched) | 72 |
| #21 | single demyelinating event:ti,ab,kw (Word variations have been searched) | 9 |
| #22 | #5 or #6 or #7 or #8 or #9 or #10 or #11 or #12 or #13 or #14 or #15 or #16 or #17 or #18 or #19 or #20 or #21 | 1436 |

All Results (1436)

Cochrane Reviews (41)

Other Reviews (8)

Trials (1369)

Methods Studies (4)

Technology Assessments (6)

Economic Evaluations (8)

Cochrane Groups (0)

### Science Citation Index (Web of Knowledge), searched 10/02/2016

| # 19 | [1,030](http://apps.webofknowledge.com/summary.do?product=WOS&doc=1&qid=29&SID=Z1q4pyyjwu46FhJgpOW&search_mode=AdvancedSearch&update_back2search_link_param=yes) | #17 NOT #18  Indexes=SCI-EXPANDED Timespan=All years |
| --- | --- | --- |
| # 18 | [93](http://apps.webofknowledge.com/summary.do?product=WOS&doc=1&qid=27&SID=Z1q4pyyjwu46FhJgpOW&search_mode=AdvancedSearch&update_back2search_link_param=yes) | (#17) AND DOCUMENT TYPES: (Meeting Abstract OR Proceedings Paper)  Indexes=SCI-EXPANDED Timespan=All years |
| # 17 | [1,123](http://apps.webofknowledge.com/summary.do?product=WOS&doc=1&qid=26&SID=Z1q4pyyjwu46FhJgpOW&search_mode=CombineSearches&update_back2search_link_param=yes) | #16 OR #15  Indexes=SCI-EXPANDED Timespan=All years |
| # 16 | [122](http://apps.webofknowledge.com/summary.do?product=WOS&doc=1&qid=25&SID=Z1q4pyyjwu46FhJgpOW&search_mode=CombineSearches&update_back2search_link_param=yes) | #14 AND #10  Indexes=SCI-EXPANDED Timespan=All years |
| # 15 | [1,039](http://apps.webofknowledge.com/summary.do?product=WOS&doc=1&qid=23&SID=Z1q4pyyjwu46FhJgpOW&search_mode=CombineSearches&update_back2search_link_param=yes) | #11 AND #10  Indexes=SCI-EXPANDED Timespan=All years |
| # 14 | [216,848](http://apps.webofknowledge.com/summary.do?product=WOS&doc=1&qid=22&SID=Z1q4pyyjwu46FhJgpOW&search_mode=CombineSearches&update_back2search_link_param=yes) | #13 OR #12  Indexes=SCI-EXPANDED Timespan=All years |
| # 13 | [167,718](http://apps.webofknowledge.com/summary.do?product=WOS&doc=1&qid=21&SID=Z1q4pyyjwu46FhJgpOW&search_mode=AdvancedSearch&update_back2search_link_param=yes) | TS=(metaanalys* or meta-analys* or (meta NEAR/1 analys*))  Indexes=SCI-EXPANDED Timespan=All years |
| # 12 | [80,440](http://apps.webofknowledge.com/summary.do?product=WOS&doc=1&qid=20&SID=Z1q4pyyjwu46FhJgpOW&search_mode=AdvancedSearch&update_back2search_link_param=yes) | TS=(systematic* NEAR/1 review*)  Indexes=SCI-EXPANDED Timespan=All years |
| # 11 | [1,393,569](http://apps.webofknowledge.com/summary.do?product=WOS&doc=1&qid=19&SID=Z1q4pyyjwu46FhJgpOW&search_mode=AdvancedSearch&update_back2search_link_param=yes) | TS=(random* or (clinical NEAR/1 trial*) or (controlled NEAR/1 trial*) or rct)  Indexes=SCI-EXPANDED Timespan=All years |
| # 10 | [16,869](http://apps.webofknowledge.com/summary.do?product=WOS&doc=1&qid=17&SID=Z1q4pyyjwu46FhJgpOW&search_mode=CombineSearches&update_back2search_link_param=yes) | #9 OR #8 OR #7 OR #6 OR #5 OR #4 OR #3 OR #2 OR #1  Indexes=SCI-EXPANDED Timespan=All years |
| # 9 | [96](http://apps.webofknowledge.com/summary.do?product=WOS&doc=1&qid=16&SID=Z1q4pyyjwu46FhJgpOW&search_mode=AdvancedSearch&update_back2search_link_param=yes) | TS="first demyelinating event"  Indexes=SCI-EXPANDED Timespan=All years |
| # 8 | [1,195](http://apps.webofknowledge.com/summary.do?product=WOS&doc=1&qid=15&SID=Z1q4pyyjwu46FhJgpOW&search_mode=AdvancedSearch&update_back2search_link_param=yes) | TS="clinically isolated syndrome"  Indexes=SCI-EXPANDED Timespan=All years |
| # 7 | [687](http://apps.webofknowledge.com/summary.do?product=WOS&doc=1&qid=13&SID=Z1q4pyyjwu46FhJgpOW&search_mode=AdvancedSearch&update_back2search_link_param=yes) | TS="ADEM"  Indexes=SCI-EXPANDED Timespan=All years |
| # 6 | [462](http://apps.webofknowledge.com/summary.do?product=WOS&doc=1&qid=10&SID=Z1q4pyyjwu46FhJgpOW&search_mode=AdvancedSearch&update_back2search_link_param=yes) | TS="devic"  Indexes=SCI-EXPANDED Timespan=All years |
| # 5 | [1,596](http://apps.webofknowledge.com/summary.do?product=WOS&doc=1&qid=9&SID=Z1q4pyyjwu46FhJgpOW&search_mode=AdvancedSearch&update_back2search_link_param=yes) | TS=("acute disseminated" NEAR/1 encephalomyelitis)  Indexes=SCI-EXPANDED Timespan=All years |
| # 4 | [3,531](http://apps.webofknowledge.com/summary.do?product=WOS&doc=1&qid=8&SID=Z1q4pyyjwu46FhJgpOW&search_mode=AdvancedSearch&update_back2search_link_param=yes) | TS="neuromyelitis optica"  Indexes=SCI-EXPANDED Timespan=All years |
| # 3 | [4,584](http://apps.webofknowledge.com/summary.do?product=WOS&doc=1&qid=7&SID=Z1q4pyyjwu46FhJgpOW&search_mode=AdvancedSearch&update_back2search_link_param=yes) | TS="optic neuritis"  Indexes=SCI-EXPANDED Timespan=All years |
| # 2 | [1,699](http://apps.webofknowledge.com/summary.do?product=WOS&doc=1&qid=6&SID=Z1q4pyyjwu46FhJgpOW&search_mode=AdvancedSearch&update_back2search_link_param=yes) | TS=(transverse NEAR/1 myelitis)  Indexes=SCI-EXPANDED Timespan=All years |
| # 1 | [6,786](http://apps.webofknowledge.com/summary.do?product=WOS&doc=1&qid=14&SID=Z1q4pyyjwu46FhJgpOW&search_mode=AdvancedSearch&update_back2search_link_param=yes) | TS=(demyelinating NEAR/2 (disease* OR disorder*))  Indexes=SCI-EXPANDED Timespan=All years |

### Cochrane MS group register of trials, searched 26/02/2016

Keywords for CIS

{interferon\*} OR {interferon beta} OR {beta-1 interferon} OR {beta 1 interferon} OR {interferon beta-1\*} OR {rebif} OR {avonex} OR {Betaseron} OR {beta-seron} OR {betaferon} OR {beta-IFN-1\*} OR {interferon beta-1\*} OR {Interferon-beta\*} OR {interferon beta\*} OR {recombinant interferon beta-1\*} OR {beta-1a interferon} OR {beta 1a interferon} OR {interferon beta-1a} OR {beta 1b interferon} OR {interferon beta1b } OR {IFNb-1b} OR {IFNbeta-1b} OR {interferon beta-1b} OR {copolymer-1} OR {cop-1} OR {copaxone} OR {glatiramer acetate} OR {cpx} OR {cop1} OR {copolymer} OR {glatiramer} OR {polyethylene glycol-interferon-beta-1a} OR {PEG IFN-beta-1a} OR {Pegylated interferon beta-1a} OR {Ocrelizumab}

AND

clinically isolated syndrome* OR first demyelinating event* OR first demyelinating episode OR first demyelinating attack OR First event OR first episode OR first clinical episode OR single clinical episodes OR first demyelinating event/* OR clinically isolated syndrome*

Total: 188

## Multiple Sclerosis searches

Full search strategies for the MS searches are available in the report of the larger project (*Melendez-Torres GJ, Auguste P, Armoiry X, et al. (2017) Clinical effectiveness and cost-effectiveness of beta-interferon and glatiramer acetate for treating multiple sclerosis: systematic review and economic evaluation. Health technology assessment (Winchester, England) 21 (52):1-352. doi:10.3310/hta21520*)

## Additional searches for both Multiple Sclerosis and Clinically Isolated Syndrome

### ClinicalTrials.gov, searched 03/05/2016

Advanced Search

182 studies found for: Interventional Studies | multiple sclerosis OR clinically isolated syndrome OR CNS demyelinating OR transverse myelitis OR neuromyelitis optica | interferon OR glatiramer OR betaferon OR betaseron OR avonex OR plegridy OR rebif OR extavia OR copaxone | Phase 2, 3, 4

*WHO ICTRP, searched 14/07/2016*

(Relapsing Remitting Multiple Sclerosis OR RRMS OR clinically isolated syndrome OR CNS demyelinating OR transverse myelitis OR neuromyelitis optica) in the Condition

AND

(interferon OR glatiramer OR betaferon OR betaseron OR avonex OR plegridy OR rebif OR extavia OR copaxone) in the Intervention

588 records for 175 trials found

*Websites*

|  | **Name (Brand)** | **Website address** | **Date searched** |
| --- | --- | --- | --- |
| **Companies sponsors** | Bayer (BETAFERON) | http://www.bayer.co.uk/  http://pharma.bayer.com/ | 26/04/2016 |
|  | Biogen Idec (AVONEX and PLEGRIDY) | https://www.biogen-international.com/  https://www.biogen.uk.com/ | 28/04/2016 |
|  | Merck Serono (REBIF) | http://biopharma.merckgroup.com/en/index.html |  |
|  | Novartis (EXTAVIA) | https://www.novartis.com  https://www.novartis.co.uk/ | 28/04/2016 |
|  | Teva Pharmaceuticals (COPAXONE) | http://www.tevapharm.com/research_development/  http://www.tevauk.com/ | 01/05/2016 |
| **Patient carer groups** | Brain and Spine Foundation | http://www.brainandspine.org.uk | 01/05/2016 |
|  | Multiple Sclerosis National Therapy Centres | http://www.msntc.org.uk | 01/05/2016 |
|  | MS UK | http://www.ms-uk.org | 01/05/2016 |
|  | Multiple Sclerosis Society | https://www.mssociety.org.uk | 01/05/2016 |
|  | Multiple Sclerosis Trust | https://www.mstrust.org.uk | 01/05/2016 |
|  | Neurological Alliance | http://www.neural.org.uk | 01/05/2016 |
|  | The Brain Charity (formally known as Neurosupport) | http://www.thebraincharity.org.uk | 01/05/2016 |
|  | Sue Ryder | http://www.sueryder.org | 01/05/2016 |
| **Professional groups** | Association of British Neurologists | http://www.theabn.org | 01/05/2016 |
|  | British Neuropathological Society | http://www.bns.org.uk | 01/05/2016 |
|  | Institute of Neurology | https://www.ucl.ac.uk/ion  https://www.ucl.ac.uk/ion/departments/neuroinflammation  http://discovery.ucl.ac.uk | 01/05/2016 05/05/2016 10/05/2016 |
|  | Primary Care Neurology Society | http://www.p-cns.org.uk | 01/05/2016 |
|  | Therapists in MS | https://www.mstrust.org.uk/health- professionals/professional-networks/ therapists-ms-tims/research | 01/05/2016 |
|  | United Kingdom Multiple Sclerosis Specialist Nurse Association | http://www.ukmssna.org.uk | 01/05/2016 |
| **Relevant research groups** | Brain Research Trust | http://www.brt.org.uk/research | 01/05/2016 |
|  | British Neurological Research Trust | http://www.ukscf.org  http://www.ukscf.org/about-us/ bnrt.html | 01/05/2016 |
|  | Cochrane Multiple Sclerosis and Rare Diseases of the Central Nervous System | http://www.cochranelibrary.com  http://msrdcns.cochrane.org/our-reviews | 01/05/2016 |
|  | National Institute for Health Research | http://www.nihr.ac.uk/research/  http://www.nihr.ac.uk/industry/  http://www.nihr.ac.uk/policy-and-standards/ | 01/05/2016 |

| Study, Active treatment | DBP / OLE duration (years) | **Early DMT**  **(*patients originally randomised to active treatment*)** | | | | | **Delayed DMT**  **(*patients originally randomised to placebo*)** | | | | | Risk of CDMS – adjusted  HR (95% CI) | Risk of disease progression  HR (95% CI) | p (disability progression) | p  (ARR) |
| --- | --- | --- | --- | --- | --- | --- | --- | --- | --- | --- | --- | --- | --- | --- | --- |
|  |  | Patients entering OLE  n (%) | Patients developing CDMS at max FUP (%) | Mean (SD) change in EDSS (baseline to end of FUP) | Confirmed disability progression (%) | ARR | Patients entering OLE  n (%) | Patients developing CDMS at max FUP (%) | Mean (SD) change in EDSS (baseline to end of FUP) | Confirmed disability progression (%) | ARR |  |  |  |  |
| PRECISE[27], GA | 3/2 | 198 (81) | 29.4 | 0.12 (1.04) | 20.5 | 0.11 | 211 (89) | 46.5 | 0.06 (0.90) | 21.4 | 0.16 | 0.59 (0.44–0.80) | na | NS | 0.024 |
| CHAMPS[24,25], IFN beta-1a IM | 2/3 | 100 (52) | 36 | na | na | 0.17± 0.24 | 103 (54) | 49 | na | na | 0.32 ± 0.51 | 0.57 (0.38-0.86) | na | na | 0.02 |
|  | 2/8 |  | 58 | na | na | 0.16± 0.18 |  | 69 | na | na | 0.33 ± 0.041 | 0.61 (0.45-0.82) | na | na | 0.02 |
| BENEFIT[20-23], IFN beta-1b SC | 2/1 | 261 (90) | 37 | na | 16 | 0.23 | 157 (89) | 51 | na | 24 | 0.3 | 0.59 (0.44-0.80) | 0.60 (0.39-0.92) | 0.022 | 0.029 |
|  | 2/3 |  | 46 | - 0.03 (1.19) | 25 | 0.21 |  | 57 | 0.07 (1.08) | 29 | 0.27 | 0.63 (0.48-0.83) | 0.76 (0.50-1.17) | 0.177 | 0.014 |
|  | 2/6 |  | 55 | 0.38 (1.22) | 22.5 | 0.196 |  | 66 | 0.07 (1.11) | 24.5 | 0.255 | 0.67 (0.53-0.88) | na | na | 0.0012 |
|  | 2/9 |  | na | 0.55 (1.52) | 18.6 | 0.21 |  | na | 0.72 (1.41) | 24.3 | 0.26 | 0.67 (0.53-0.85) | na | na | 0.0018 |
| REFLEX[28,26], IFN beta-1a SC | 2/1 | 127 (74) | 27.1 | na | 13.2 | na | 133 (78) | 41.3 | na | 7.5 | na | 0.55 (0.38 - 0.82) | na | 0.205 | / |
|  | 2/3 |  | 39.2 | -0.05 | 18.4 |  |  | 44.6 | -0.16 | 11 |  | 0.683(0.48-0.98) |  | / |  |

Online resource 2- Main outcomes of open-label extension studies comparing early versus delayed DMT

**Online resource 3: risk of bias by study**

**Online resource 4: risk of bias across domains**
